# Supplementary material for: Reliable interpretability of biology-inspired deep neural networks
Source: NPJ Syst Biol Appl. 2023 Oct 10;9:50. doi: 10.1038/s41540-023-00310-8 (PMC10564878; doi:10.1038/s41540-023-00310-8)
Supplement: Supplementary file 1 — Supplementary Figures 1–6 [file 41540_2023_310_MOESM1_ESM.pdf]

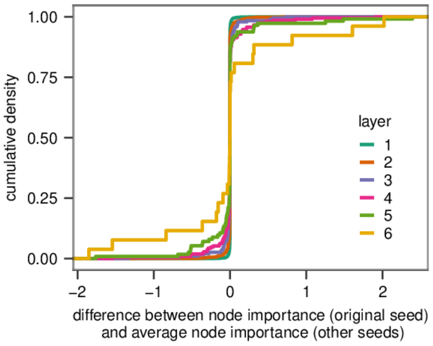

Supplementary Figure 1. Distribution of the differences between node importance scores obtained using the original seed and the average node importance seed across the 50 replicate networks (n = 11,147 differences).

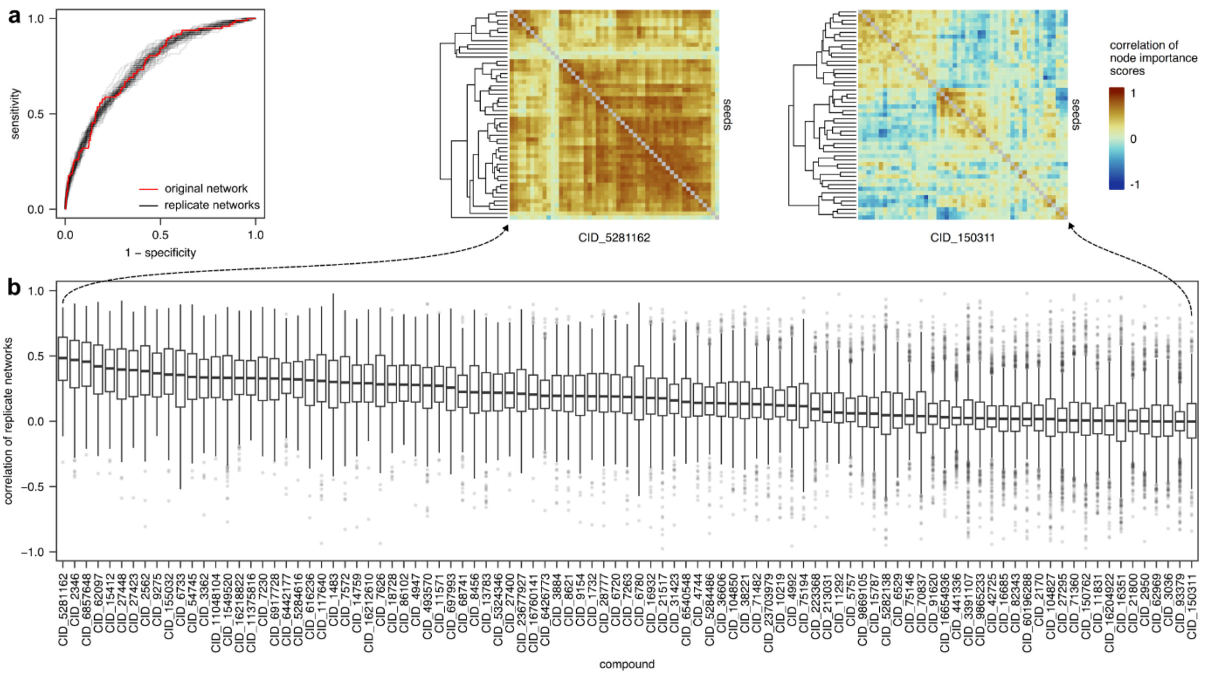

Supplementary Figure 2. Analysis of robustness in the DTox model[23]. DTox predicts toxicity (output labels) of compounds (observations) from their structural properties (input features) in a network constructed from Reactome pathways, providing interpretations at the level of proteins and pathways. Importantly, interpretations of DTox are local interpretations in that one set of importance scores is obtained for every compound (observation) and trained replicate network. (a) ROC curves of replicate DTox networks (n = 51 replicate networks), showing that prediction performance is comparable between replicates. (b) Pairwise correlations (Pearson's R) between replicate networks across importance scores obtained from DTox, showing a wide range of correlations and thus a limited robustness for a large number of compounds (n = 1,275 correlation values per compound). For the compounds with the highest and lowest median correlation, heatmaps depict correlations (Pearson's R) of the replicate networks. Boxes display the median value and 25 and 75% quartiles; the whiskers are extended to the most extreme value inside the 1.5-fold interquartile range; dots show outliers.

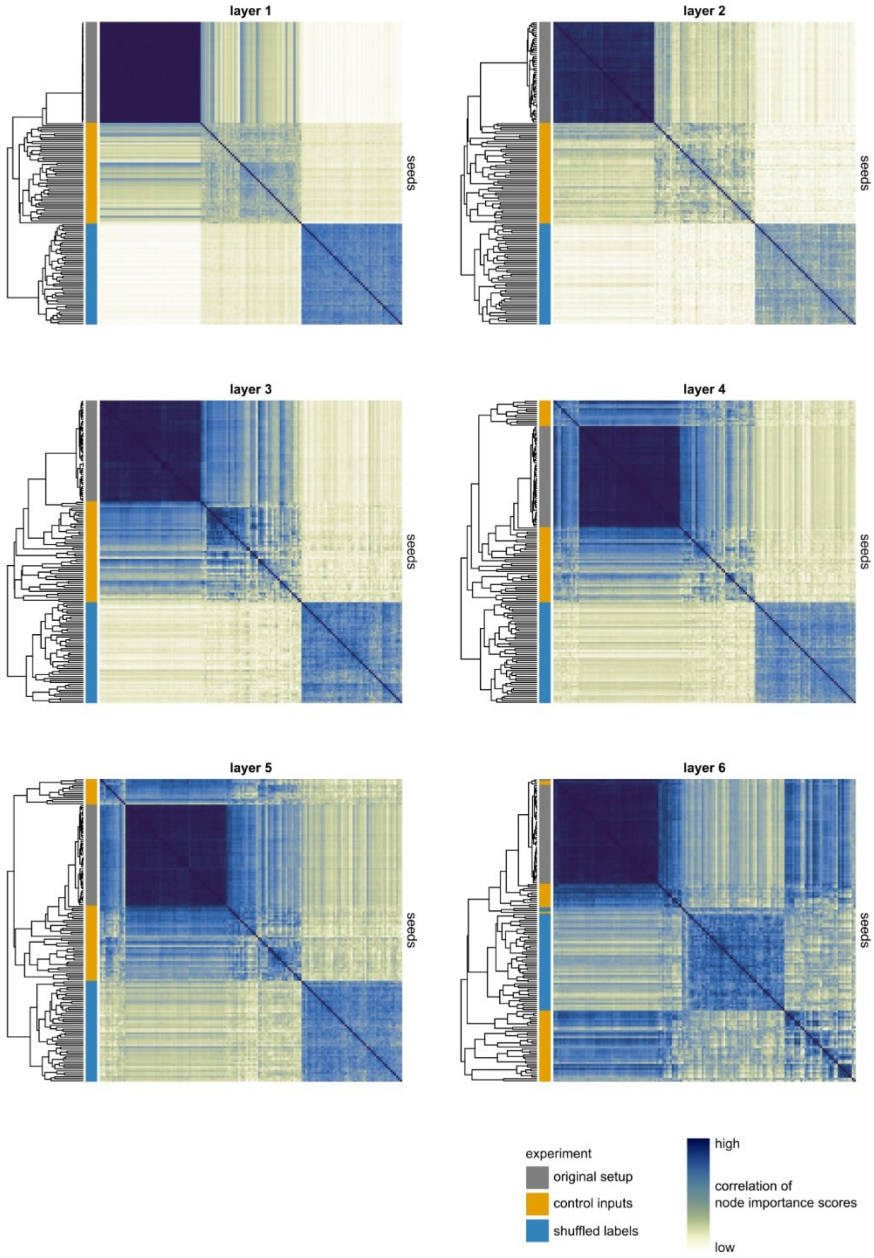

Supplementary Figure 3. Layer-wise correlation (Pearson's R) of node importance scores across experimental approaches. Each of the 103 rows and columns (51 seeds x 3 setups) represents a different network.

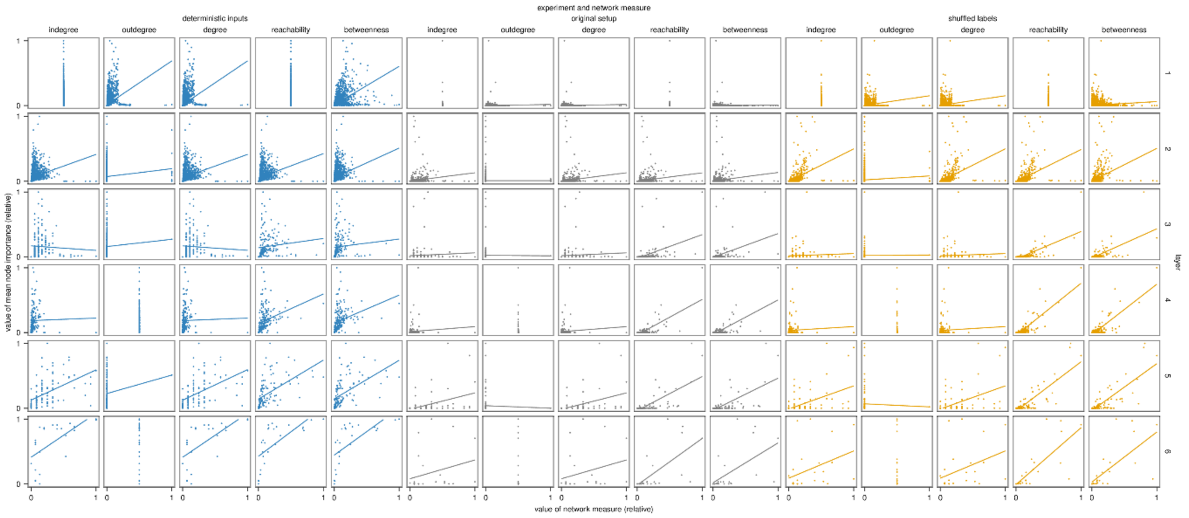

Supplementary Figure 4. Scatterplots for all cells of the heatmap in **Figure 5a.** Each point corresponds to a hidden node (n = 9229, 1387, 193, 210, 113, and 26 in each panel of layers 1 to 6, respectively).

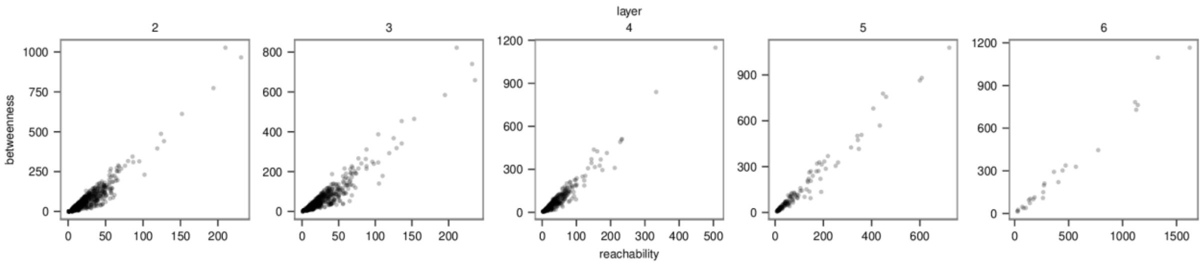

Supplementary Figure 5. Scatterplot of betweenness and reachability in each P-NET layer. The subplot for layer 1 (gene layer) is omitted, since all nodes in this layer share a reachability of 1.

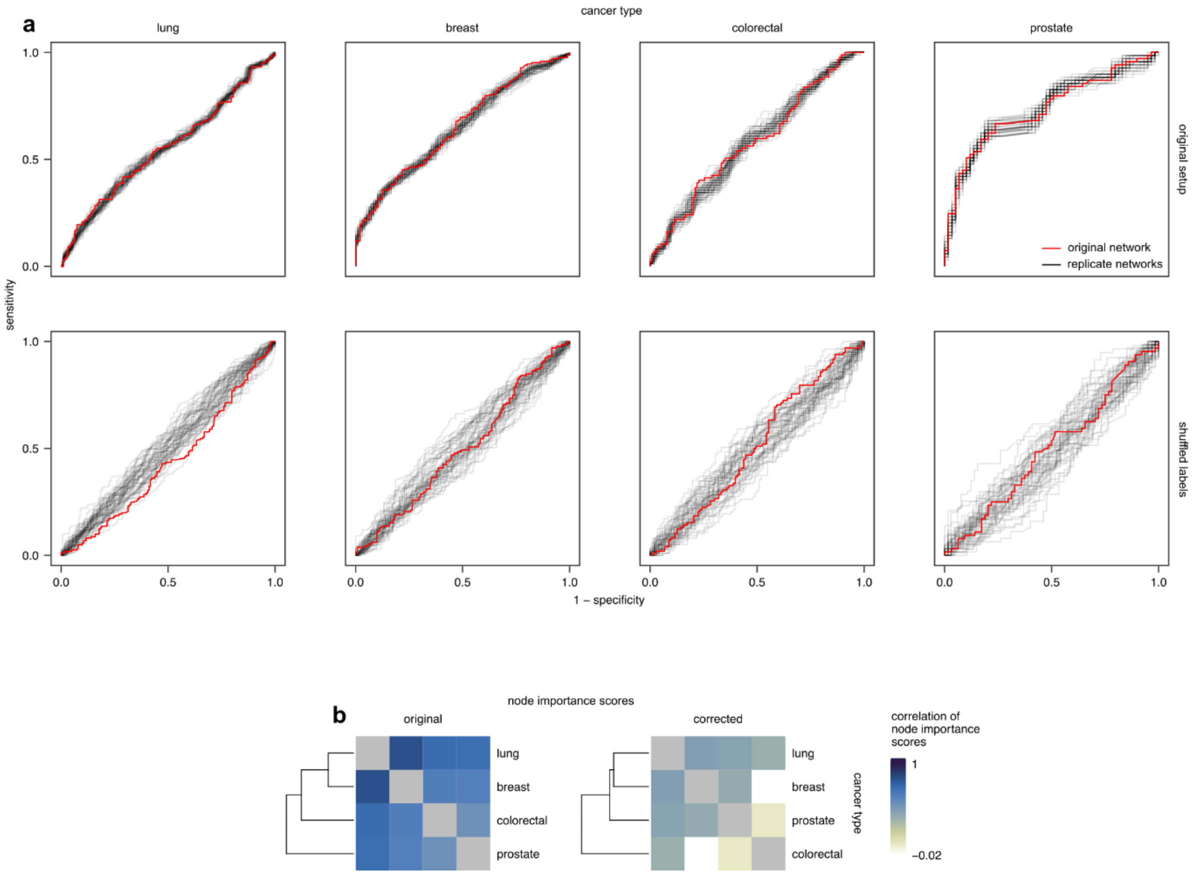

Supplementary Figure 6. Correction approach on MSK-IMPACT data. (a) ROC curves of P-NET trained on subsets (i.e., different cancer types) of the MSK-IMPACT 2017 dataset (n = 51 replicate networks per experiment). ROC curves are shown for the original setup (top) and shuffled labels (bottom). (b) Correlation (Pearson's R) heatmap showing correlations of average node importance scores from the original setup (left) and after the correction (right) using differential importance scores.
